# Veterinary Department of McGill University

**Published:** 1898-07

**Authors:** 


### VETERINARY DEPARTMENT OF McGILL UNIVERSITY.

The Montreal Veterinary College was established in 1866, and the session of 1898–1899 will convene its thirty-third session, becoming affiliated with McGill University with the session of 1889–1890.

This college instituted a three-years' course of six months each as its curriculum in 1877, and has maintained these requirements ever since. It can be said that this college was the pioneer in higher veterinary education. Its entrance examination is rigidly enforced and continues to be advanced higher and higher. Rudiments of Latin as a necessary part of matriculation will be shortly required. This accounts largely for the uniform high standing of the graduates of this school, many of whom have been selected as teachers in other veterinary colleges, notably Veterinary Department of University of Pennsylvania, American Veterinary College, Chicago Veterinary College, California Veterinary College, New York State Veterinary College, United States College of Veterinary Surgeons, National Veterinary College, Amherst Agricultural College, and French Veterinary School of Montreal. By virtue of a money grant, thirteen free scholarships are annually awarded to the Province of Quebec. Greatly increased laboratory facilities and advantages were obtained by affiliation. The examinations held regularly determine the advancement of the students. The school has graduated two hundred and fifty-two veterinarians.

